# Epstein-Barr virus (EBV) serology and its impact on oral human papillomavirus (HPV) infection outcomes in children during early childhood

**DOI:** 10.1128/spectrum.00071-25

**Published:** 2025-08-01

**Authors:** Sanni Rinne, Birgitta Michels, Julia Butt, Kari Syrjänen, Seija Grenman, Tim Waterboer, Stina Syrjänen, Karolina Louvanto

**Affiliations:** 1 Department of Obstetrics and Gynecology, Faculty of Medicine and Health Technology, Tampere University 101287 , Tampere, Finland; 2 German Cancer Research Center (DKFZ) 28333, Heidelberg, Germany; 3 SMW Consultants, Ltd., Kaarina, Finland; 4 Department of Obstetrics and Gynecology, Turku University Hospital, University of Turku 541620 , Turku, Finland; 5 Department of Oral Pathology, Institute of Dentistry, Faculty of Medicine, University of Turku 169296, Turku, Finland; 6 Department of Pathology, Turku University Hospital, Turku, Finland; 7 Department of Obstetrics and Gynecology, Tampere University Hospital 60670, Tampere, Finland; University of Manitoba, Winnipeg, Manitoba, Canada

**Keywords:** human papillomavirus, oral HPV infection, children, Epstein-Barr virus, EBV serology

## Abstract

Epstein-Barr virus (EBV) and various human papillomaviruses (HPVs) commonly infect the oral mucosa, yet the longitudinal effects of these infections and their potential coinfections remain poorly understood. This study investigated whether early EBV infection and antibody responses influence oral HPV infections in young children. We included 283 children from the Finnish Family HPV cohort study, who were followed for 3 years post-birth. Oral and blood samples were collected at six time points (1, 2, 6, 12, 24, and 36 months). HPV genotyping was performed with Luminex and EBV-IgG antibodies to Zebra, early antigen-diffuse (EA-D), EBV nuclear antigen 1, and viral capsid antigen p18 with fluorescent bead-based multiplex serology. We noticed that most children (91.4%; *n* = 254) experienced the vanishing of maternal EBV-IgG antibodies within 11.3 months, and by 36 months, 17% (41/238) of the children had developed their own EBV antibodies. Intriguingly, higher paternal education levels were strongly associated with lower EBV seropositivity in children at ages 2 and 3, with an odds ratio(OR) range of 0.06 to 0.16 (95% confidence interval range 0.005–0.91). Additionally, children with the highest baseline titers of EA-D antibodies had 2.5- and threefold risk for incident oral HPV infection and its clearance, respectively. Our findings suggest that EBV seropositivity at 3 years of age is relatively low in our Finnish data, and the level of paternal education was a significant protective factor against early EBV seropositivity. Moreover, the observed association between high EA-D antibody titers and oral HPV infection underscores the need for further research into the complex interactions between EBV and HPV.

**IMPORTANCE:**

Epstein-Barr virus (EBV) and human papillomaviruses (HPVs) are known to cause cancers in the head and neck region, yet their interactions in young children remain largely unexplored. EBV, associated with infectious mononucleosis, and oral HPV, often asymptomatic in early childhood, target similar anatomical regions but are poorly studied together in this age group. Understanding these interactions is crucial, as the incidence of HPV-related oropharyngeal cancers has been rising over recent decades, making the natural history of oral HPV infections a critical research focus. While our study found no significant link between EBV seropositivity and oral HPV outcomes in children, evidence in adults suggests these viruses may interact in cancer development. Investigating this dynamic in early childhood could provide valuable insights into infection patterns and inform prevention strategies to reduce cancer risks later in life.

## INTRODUCTION

Epstein-Barr virus (EBV) and human papillomavirus (HPV) are both widespread oncogenic viruses acquired early in life, often without symptoms. EBV, a member of the Herpesviridae family, establishes a lifelong latency in B lymphocytes following primary infection of the oropharyngeal oral mucosal cells (1). The clinical presentation of acute EBV infection varies and includes both symptomatic and asymptomatic infections: infectious mononucleosis (IM), most typical in adolescence, and asymptomatic infections, which are more typical in childhood (2, 3). Notably, children may have a less efficient antibody response during acute infections and may present with atypical symptoms such as runny nose, diarrhea, and rashes (4–6). Children do not usually develop severe symptoms from IM; however, having an early-life infection could increase the risk of EBV-related malignancies (7) such as Burkitt’s and Hodgkin’s lymphomas as well as nasopharyngeal and gastric carcinomas (NPCs) (8). Studies on early-life infections and the risk of EBV-related malignancies have primarily focused on Burkitt’s lymphoma (9, 10) and NPCs (11, 12). The estimated seroprevalence of EBV among children ranges from 20% to 80%, varying between the geographic location and age (2).

The diagnosis of EBV is made via serological tests: EBV-IgM antibodies detect primary infections, and past infections or reactivation of EBV can be detected by EBV-IgG antibodies (2, 13–15). Most diagnostically used antibodies are EA-D (early antigen-diffuse) IgG, EBNA-1 (EBV nuclear antigen 1) IgG, VCA (viral capsid antigen) IgG, and IgM antibodies (6, 13). The IgG antibodies to these antigens have also been linked to being markers for EBV-related malignancies in addition to the antibodies of the Zebra (transcriptional transactivator protein) antigen (16–18).

Persistent high-risk HPVs (HR-HPVs) are well-known for their role in cervical cancer and its precursors, but they also contribute to anogenital and head and neck cancers (head and neck squamous cell carcinomas) (19). In childhood, HPV typically manifests as cutaneous warts or mucosal papillomas which are induced by low-risk HPV infections (20), yet oral infections by HR-HPVs have also been detected (21). Oral HPV DNA prevalence in children aged 0–18 years has varied from 0 to 47% in previous studies (21–30). In children under the age of 7, the prevalence has been reported to vary from 0% to 20% (23, 25–30). The true meaning of early-life infections remains unclear; however, it is thought that most infections are likely to clear as the child grows older (31). On the contrary, there is emerging evidence that early-life infections can increase the risk for infection-related cancers later in life (7). Therefore, more knowledge on co-factors leading to persistent oral HPV infections already in early childhood is crucially needed.

While EBV and HPV are known to infect similar cells at similar anatomical sites, earlier literature on evaluating the co-occurrence of the two viruses in children is scarce. In adults, the two viruses have been found simultaneously in cancerous tissues, especially in oropharyngeal cancers (32–34), and it has been speculated that other cancerous pathogens like EBV could be potential co-factors in HPV pathogenesis (35). This study aimed to further explore the possible association between the different EBV antigens and serological response and their impact on oral HPV infections among children during their first 3 years of life.

## MATERIALS AND METHODS

### The Finnish Family HPV Study

The Finnish Family HPV Study is a longitudinal cohort study conducted jointly since 1998 at the Department of Obstetrics and Gynecology, Turku University Hospital, and the Department of Oral Pathology, Institute of Dentistry, Faculty of Medicine, University of Turku, Finland. The original study was designed to explore the dynamics of HPV infections among family members (29, 36, 37). The families were recruited in the final trimester of the mother’s pregnancy, at a minimum of 36 weeks without prior information on the oral or genital HPV status of the participants. The study included 329 mothers, 131 men, and 331 children who were followed for 36 months. A questionnaire concerning sexual habits and overall health was provided to the parents at baseline. The participants’ oral, genital, and serological status has been previously reported (38–40). The transmission of HPV between the spouses as well as between the parent and the child has also been previously determined (41).

### Sample collection

Oral scrapings from the buccal mucosa were taken with a brush (Cytobrush, MedScan, Malmö, Sweden) after birth and additionally, at 1-, 2-, 6-, 12-, 24-, and 36-month follow-up visits as described earlier (36). Blood samples for serological analyses were taken at 1-, 2-, 6-, 12-, 24-, and 36-month follow-up visits as described earlier (37).

### HPV genotyping

The HPV genotyping for oral scrapings was done using the Luminex HPV genotyping kit (Multimetrix; Progen Biotechnik GmbH) with modifications, as described earlier (42). A total of 24 different HPV genotypes were identified: six low-risk: HPV 6, 11, 42, 43, 44, and 70; and 18 high-risk: HPV 16, 18, 26, 31, 33, 35, 39, 45, 51, 52, 53, 56, 58, 59, 66, 68, 73, and 82). The children’s oral HPV DNA status during the first 6 years of life has been published earlier (28) and the data are used here in connection with EBV serology.

### EBV serology

The serum IgG antibodies of the antigens Zebra, EA-D, EBNA-1, and VCAp18 were analyzed with fluorescent bead-based multiplex serology at the German Cancer Research Center (DKFZ), Heidelberg, Germany (43). The serological samples were tested in a single round of testing (43). Seroconversion was determined by previously set medium fluorescence intensity(MFI) cut-off of 176 for Zebra, 367 for EA-D, 1,500 for EBNA-1, and 1,802 for VCAp18. The requirement for overall EBV seropositivity was having the antibodies of two or more antigens out of four above the defined MFI cut-off values, as previously described (43).

### Statistical analyses

The final study included 283 children with EBV-IgG serology available from at least three out of the six visits. A major part of the children (39.2%; *n* = 111) had data from six follow-up visits, 96 (33.9%) children had data from five follow-up visits, 55 (19.4%) children had data from four follow-up visits, and only 21 (7.4%) children had data from at least three follow-up visits. Participants with data from only the first 6 months (*n* = 2), and those with missing data from the same time (first three follow-up visits, [*n* = 3]), were excluded from the serological outcome analyses. EBV serology outcomes were grouped into (i) always seronegative: MFI values all below the MFI cut-off the whole follow-up; (ii) seroconversion: defined as an initial MFI value below the cut-off, followed by a subsequent value above the cut-off, with at least a twofold increase in MFI levels between these points; (iii) antibody fluctuation; (iv) serological decay: initial MFI value above the cut-off, followed by a subsequent value below the cut-off that stayed negative the rest of the follow-up; and (v) seropersistence: all visits seropositive.

Different EBV antigens (Zebra, EA-D, EBNA-1, and VCAp18) were evaluated individually and in combinations. Correlations between seroreactivity to four EBV antigens at each time point and maternal corresponding antibody levels at baseline were analyzed using the Spearman rank correlation coefficient. Correlations were classified as low (coefficient of 0.00–0.40), moderate (0.40–0.60), and high (0.60–1.00). To measure the strengths of associations between different variables, including risk factors and oral HPV infection outcomes, univariate unconditional logistic regression was used. For oral HPV infection outcomes, the positive antibody levels of EBV were divided into tertiles (low, middle, and high tertiles). All statistical analyses were performed using Stata 16.1 (STATA Corp., TX), and *P*-values were two-sided, and <0.05 were considered statistically significant.

## RESULTS

**Fig 1:**
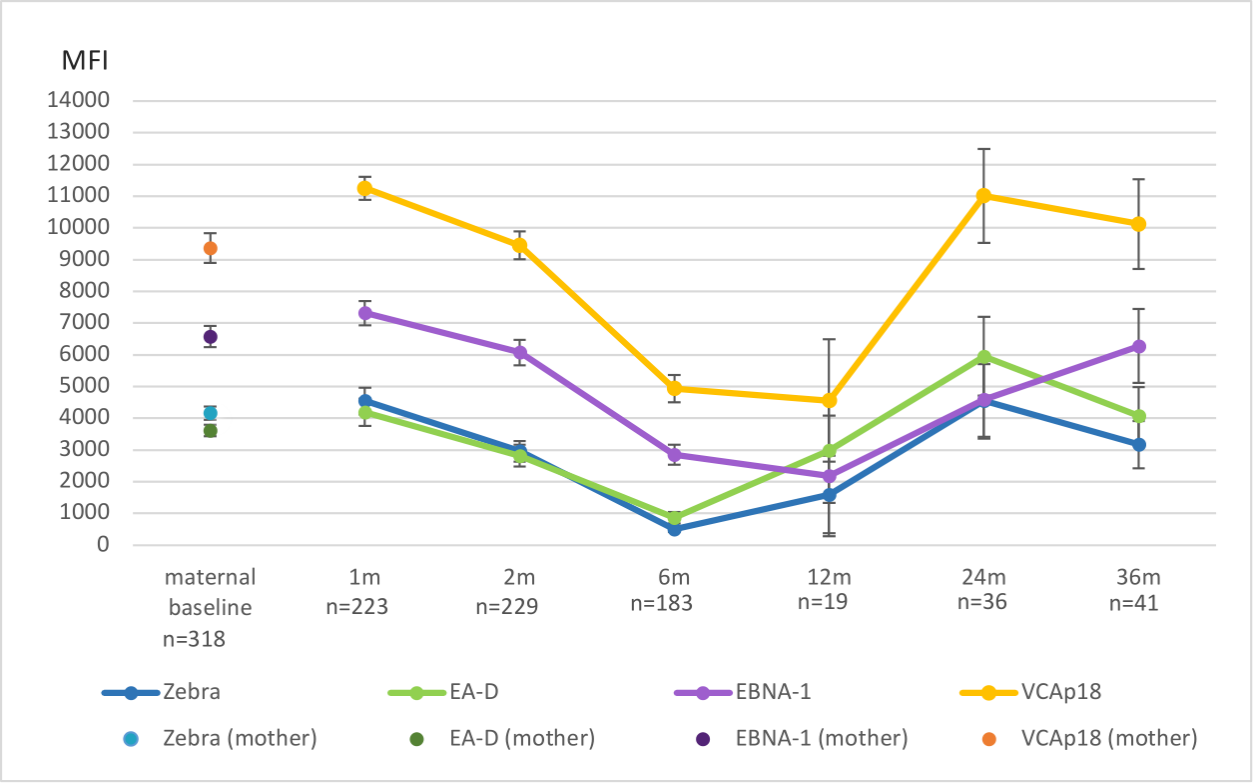
Mean levels of EBV-IgG antibodies to individual EBV antigens (Zebra, EA-D, EBNA-1, and VCAp18) in children who were EBV-seropositive at each follow-up visit during the 36-month follow-up period. The number of seropositive children at each visit is indicated on the *x*-axis. For comparison, maternal IgG antibody levels to the same individual EBV antigens measured during the 3rd trimester are also shown. Error bars represent 95% confidence intervals of the mean antibody levels at each time point.

Most of the children, 97.3% (*n* = 223), were overall EBV-seropositive at baseline (1-month visit) (Fig. 1). Strong correlations were observed between the mother’s four EBV-IgG antibody levels (Zebra, EA-D, EBNA-1, and VCAp18) during the third trimester and her offspring’s corresponding titers at the 1- and 2-month visits (*r* = 0.60–0.91, *P* < 0.001) (Table 1). This correlation remained statistically significant until the 6-month visit (0.19–0.24, *P* < 0.05). All maternal EBV antibodies to Zebra, EA-D, EBNA-1, and VCAp18 vanished by the 6- and/or 12-month visits (mean 11.3 months), respectively.

**TABLE 1:** Bivariate correlations (Spearman’s rho) between maternal EBV-IgG antibody levels at baseline (3rd trimester) and offspring’s EBV-IgG titers by visit^*a*^^,^^*b*^

1 month	
EBNA-1	0.76**
Zebra	0.71**
EA-D	0.83**
VCAp18	0.91**
2 months	
EBNA-1	0.72**
Zebra	0.60**
EA-D	0.66**
VCAp18	0.86**
6 months	
EBNA-1	0.19*
Zebra	0.24**
EA-D	0.23**
VCAp18	0.19*
12 months	
EBNA-1	0.05
Zebra	0.07
EA-D	0.03
VCAp18	0.05
24 months	
EBNA	0.02
Zebra	0.03
EA-D	0.03
VCAp18	0.06
36 months	
EBNA	0.09
Zebra	0.04
EA-D	0.03
VCAp18	0.00

^*a*^ Titers of four EBV antigens (Zebra, EA-D, EBNA-1, and VCAp18) were analyzed.

^*b*^ Statistically significant results: * *P*<0.05; ** *P*<0.001.

**Fig 2:**
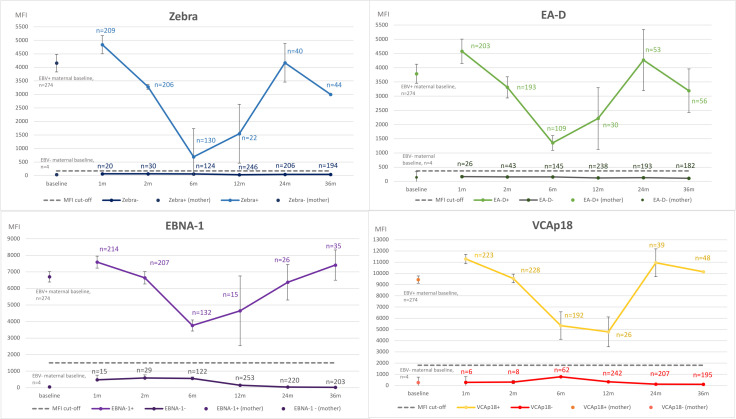
IgG antibody levels to individual EBV antigens Zebra, EA-D, EBNA-1, and VCAp18 in seropositive and seronegative children during their first 36 months of life. The number of seropositive children and their IgG antibody titers (mean with standard deviations) to individual EBV are given separately to visualize the antibody fluctuation during follow-up. For comparison, maternal IgG antibody levels to the same individual EBV antigens measured during the 3rd trimester among both seropositive and seronegative mothers are also shown. The dashed line represents the MFI cut-off for seropositivity for each antigen, individually. Error bars represent 95% confidence intervals of the mean antibody levels.

When the individual antigens Zebra-, EA-D-, EBNA-1-, and VCAp18-seropositive and -seronegative MFI levels were evaluated, these showed very similar variations as the overall EBV seropositivity (Fig. 2). In children, EBV titers to Zebra, EA-D, and EBNA-1 increased between the 6- and 24-month visit, with a decline to the 36-month visits with Zebra and EA-D, except with EBNA-1 that continued to increase. The titers to VCAp18 were lowest at the 12-month visit, followed by an increase at the 24-month and again a decline at the 36-month visit. The increase in antibody titers after the initial decrease (maternal antibody waning) is indicative of a primary EBV infection and endogenous immune response among the children of our cohort. At the 12-month follow-up, 7.1% (*n* = 19) of the children were EBV-seropositive, and at 36 months, this was up to 17.2% (*n* = 41). Of these seropositive children at the 24- and 36-month follow-up visits, 16%–18%, 21%–24%, 11%–15%, and 16%–20% were EBV-seropositive to the individual Zebra, EA-D, and EBNA-1, and VCAp18 antigens, respectively.

Most children experienced the vanishing of maternal antibodies (91.4%; *n* = 254) and remained seronegative to the end of the 3-year follow-up (79.1%; *n* = 220) (Table 2). Yet, a total of 34 (12.2%) children subsequently seroconverted, indicating a primary EBV infection within 27.2 months. The remaining children remained either always seronegative (*n* = 10), seropositive (*n* = 9), or experienced antibody fluctuation (*n* = 5).

**TABLE 2:** Serological outcomes of the children during the 36-month follow-up^*b*^ with the mean (±SD) antibody levels (MFI) of the four different EBV-specific antigens Zebra, EA-D, EBNA-1, and VCAp18^*a*^

Serological outcome	*n* (%)	MFI mean (± SD)	Mean time to (months)
Always seronegative	10 (3.6)		–
Zebra		42.0 (± 70.1)	
EA-D		101.7 (± 87.7)	
EBNA-1		138.1 (± 374.1)	
VCAp18		660.6 (± 1,754.7)	
Seroconversion^*c*^	34 (12.2)		27.2
Zebra		2,574.7 (± 3,059.8)	
EA-D		2,804.0 (± 3,335.6)	
EBNA-1		4,023.5 (± 3,749.5)	
VCAp18		7,028.8 (± 5,273.7)	
Antibody fluctuation	5 (1.8)		–
Zebra		1,766.2 (± 2,634.6)	
EA-D		1,750.8 (± 2,569.3)	
EBNA-1		2,262.3 (± 2,845.2)	
VCAp18		5,045.7 (± 4,774.1)	
Serological decay	220 (79.1)		11.5^*d*^
Zebra		1,279.1 (± 2,398.1)	
EA-D		1,320.2 (± 2,335.0)	
EBNA-1		2,576.0 (± 3,562.7)	
VCAp18		4,130.0 (± 4,944.8)	
Seropersistent	9 (3.2)		–^*e*^
Zebra		2,306.3 (± 2,567.6)	
EA-D		3,286.5 (± 2,832.9)	
EBNA-1		4,189.6 (± 3,804.8)	
VCAp18		1,0020.3 (± 4,956.2)	

^*a*^ The MFI mean (± SD) is representative of the children’s antibody levels throughout the whole follow-up period.

^*b*^ Participants with data from only the first 6 months (*n* = 2) and those with missing data from the same time (first three follow-up visits, [*n* = 3]) were excluded.

^*c*^ Includes those who experienced the vanishing of maternal antibodies and subsequently seroconverted again, due to primary infection.

^*d*^ Mean time of serological decay in those who stayed seronegative throughout the follow-up; the mean time for all participants was 11.3 months.

^*e*^ The mean time to seronegativity, seropersistence, or antibody fluctuation was not determined, as it coincided with the follow-up duration (not applicable, -).

From various co-factors evaluated, the associations for EBV seropositivity at ages 2 and 3 showed that the father’s education level significantly protected against seropositivity for each antigen and the overall EBV seropositivity (Table 3). The protective effect increased with higher levels of the father’s education, particularly among seropositivity to antigens Zebra and VCAp18, as well as overall EBV seropositivity (odds ratio (OR) range from 0.06 to 0.16; 95% CI range of 0.004–0.91). A similar protective effect was seen with EA-D and EBNA-1 antigens, OR range of 0.08 to 0.09 (95% CI range of 0.01–0.64). The mother’s education, family size, smoking, delivery mode, or allergies showed no significant association with seropositivity to individual EBV antigens or overall EBV-seropositivity.

**TABLE 3:** Potential risk factors for offspring seropositivity to individual EBV antigen (Zebra, EA-D, EBNA-1, and VCAp18) or overall EBV seropositivity at the 24- and 36-month^*b*^ follow-up visits assessed using univariate logistic regression^*a*^

Risk factors		Zebra	EA-D	EBNA-1	VCAp18	EBV+
		OR (95% CI)				
Mode of delivery (*n* = 214)	Vaginal	1.0	1.0	1.0	1.0	1.0
	Cesarean section	0.95 (0.38–2.35)	0.63 (0.25–1.63)	0.79 (0.25–2.47)	0.68 (0.24–1.88)	0.85 (0.32–2.20)
Breastfeeding for at least 6 months (*n* = 331)	Yes	0.92 (0.37–2.28)	1.00 (0.42–2.39)	0.73 (0.23–2.26)	1.10 (0.44–2.75)	1.00 (0.40–2.49)
Passive cigarette smoking (*n* = 202)	Yes	1.42 (0.63–3.19)	1.04 (0.49–2.18)	1.02 (0.41–2.59)	1.28 (0.57–2.89)	1.41 (0.61–3.26)
Atopy (*n* = 162)	Yes	1.68 (0.54–5.27)	1.40 (0.49–4.02)	2.48 (0.54–11.36)	1.67 (0.53–5.22)	1.61 (0.51–5.07)
Allergies (*n* = 163)	Yes	0.65 (0.21–2.04)	0.48 (0.13–1.70)	0.76 (0.21–2.82)	0.66 (0.21–2.06)	0.70 (0.22–2.22)
Disorders	No	1.0	1.0	1.0	1.0	1.0
	Asthma	N/C^*c*^	N/C	N/C	N/C	N/C
	Other	2.72 (0.24–31.22)	4.26 (0.26–70.27)	N/C	2.74 (0.24–31.47)	2.91 (0.25–33.47)
Family size (*n* = 213), *n*	3	1.0	1.0	1.0	1.0	1.0
	4	0.57 (0.25–1.31)	0.67 (0.31–1.48)	0.61 (0.23–1.62)	0.69 (0.30–1.62)	0.68 (0.29–1.58)
	≥5	0.36 (0.10–1.31)	0.61 (0.21–1.78)	0.36 (0.08–1.70)	0.42 (0.12–1.54)	0.45 (0.12–1.63)
Mother’s education (*n* = 185)	Basic education	1.0	1.0	1.0	1.0	1.0
	Vocational education	0.78 (0.23–2.61)	1.17 (0.33–4.19)	1.38 (0.27–7.11)	1.01 (0.28–3.60)	1.03 (0.29–3.69)
	Secondary school graduate	0.26 (0.05–1.25)	0.75 (0.17–3.22)	0.65 (0.10–4.36)	0.36 (0.07–1.86)	0.35 (0.07–1.79)
	College graduate	0.31 (0.08–1.13)	0.39 (0.10–1.55)	0.55 (0.10–3.16)	0.43 (0.11–1.69)	0.34 (0.08–1.39)
	Academic degree	N/C	0.10 (0.01–1.01)	N/C	N/C	N/C
Father’s education (*n* = 89)	Basic education	1.0	1.0	1.0	1.0	1.0
	Vocational education	**0.16 (0.03–0.91**)	0.18 (0.03–1.03)	**0.09 (0.01–0.64**)	**0.13 (0.02–0.76**)	**0.12 (0.02–0.71**)
	Secondary school graduate	0.13 (0.01–1.67)	0.19 (0.01–2.66)	0.17 (0.01–2.37)	0.13 (0.01–1.67)	0.13 (0.01–1.67)
	College graduate	**0.07 (0.01–0.52**)	**0.08 (0.01–0.60**)	N/C	**0.07 (0.01–0.57**)	**0.07 (0.01–0.52**)
	Academic degree	**0.06 (0.004–0.72**)	N/C	0.08 (0.01–1.02)	**0.06 (0.01–0.78**)	**0.06 (0.01–0.72**)

^*a*^ Significant results in bold.

^*b*^ Including those seropositive at both follow-up visits and excluding those with no data or fluctuating antibody levels, which results in different numbers of observations for the factors investigated.

^*c*^ Abbreviations: N/C, Not Calculated.

During the 36-month follow-up, a total of 96 of 283 (33.9%) of these children had an incident oral HR-HPV infection. A total of 87 (90.6%) children cleared their oral HPV infection, while 9 (9.4%) had persistent oral HPV16 or HPV18 infection lasting at least 12 months, with a mean persistence time of 22.4 months (range: 12–36 months). Children’s EBV serology at the age of 2–3 years (whether to the individual EBV antigens or overall EBV positivity) showed no association with the children’s oral HPV infection outcomes (Table 4). Interestingly, in the further evaluations, the highest tertile of baseline EA-D was significantly associated with increased risk for incident oral HPV infection, OR 2.55 (95% CI 1.13–5.79), and clearance, OR 3.17 (95% CI 1.40–7.14) (Table 5). Mid-level baseline Zebra antibody levels also increased oral HPV clearance risk with an OR of 2.76 (95% CI 1.21–6.27) but did not remain significant with the highest tertile.

**TABLE 4:** EBV-seropositive children^*a*^ with the EBV-specific antigens Zebra, EA-D, EBNA-1, and VCAp18 at the 24- and 36-month follow-up visit association with oral HPV infection outcomes during the first 3 years of life (incidence^*b*^, clearance^*c*^, and type-specific persistence^*d*^)

	Children’s oral HPV outcome		
Seropositivity to individual EBV antigens	Incidence (*n* = 107)^*b*^	Clearance (*n* = 99)^*c*^	Persistence (*n* = 12)^*d*^
Zebra (*n* = 32)	0.40 (0.15–1.09)	0.40 (0.15–1.09)	0.54 (0.06–4.58)
EA-D (*n* = 38)	0.63 (0.26–1.52)	0.58 (0.24–1.44)	1.33 (0.25–7.17)
EBNA-1 (*n* = 22)	0.45 (0.14–1.51)	0.58 (0.19–1.77)	0.96 (0.11–8.52)
VCAp18 (*n* = 30)	0.44 (0.16–1.21)	0.45 (0.16–1.23)	0.60 (0.07–5.16)
Overall EBV seropositivity^*a*^			
EBV+ (*n* = 30)	0.36 (0.13–1.04)	0.43 (0.16–1.17)	0.59 (0.07–5.02)

^*a*^ Being seropositive to at least two out of four EBV antigens.

^*b*^ HPV-positive status at any point during the follow-up period.

^*c*^ HPV-positive status followed by a negative HPV test.

^*d*^ HPV16 or HPV18 infection persisting over 12 months.

**TABLE 5:** Association between baseline EBV-seropositive children’s^*a*^ antibody levels (tertiles) for different EBV antigens (Zebra, EA-D, EBNA-1, and VCAp18) and their oral HPV infection outcomes during the first 3 years of life^*e*^

EBV antigen	Tertile	Children’s oral HPV outcome		
		Incidence (*n* = 107)^*b*^	Clearance (*n* = 99)^*c*^	Persistence (*n* = 12)^*d*^
Zebra (*n* = 209)	Low	1.0	1.0	1.0
	Mid	2.08 (0.97–4.46)	**2.76 (1.21–6.27)^*f*^**	4.50 (0.44–46.09)
	High	0.87 (0.40–1.91)	1.56 (0.69–3.52)	N/C
EA-D (*n* = 203)	Low	1.0	1.0	1.0
	Mid	2.08 (0.95–4.54)	1.70 (0.75–3.85)	1.32 (0.08–22.06)
	High	**2.55 (1.13–5.79)**	**3.17 (1.40–7.14)**	3.36 (0.29–39.28)
EBNA-1 (*n* = 214)	Low	1.0	1.0	1.0
	Mid	0.83 (0.40–1.75)	1.12 (0.50–2.49)	0.34 (0.03–3.51)
	High	0.58 (0.27–1.26)	1.23 (0.56–2.69)	0.33 (0.33–3.39)
VCAp18 (*n* = 223)	Low	1.0	1.0	1.0
	Mid	1.11 (0.53–2.33)	1.32 (0.62–2.83)	0.71 (0.11–4.54)
	High	0.92 (0.44–1.95)	1.05 (0.48–2.27)	N/C

^*a*^ Being seropositive to at least two out of four EBV antigens.

^*b*^ Positive HPV status at any point during the follow-up period (baseline negative).

^*c*^ Positive HPV status followed by a negative HPV test (stayed negative throughout the end of the follow-up).

^*d*^ HPV16 or HPV18 infection persisting 12 months or more.

^*e*^ Univariate logistic regression analysis was used to estimate ORs; MFI levels of antigen tertiles: Zebra (low = 176–3,065, mid = 3,066–5,804, high = 5,805–1,2780), EA-D (low = 376–2,701, mid = 2,702–5,599, high = 5,600–13,898), EBNA-1 (low = 1,500–6,741, mid = 6,742–8,926, high = 8,927–14,714), VCAp18 (low = 1,802–10,388, mid = 10,389–12,472, high = 12,473–19,673).

^*f*^ Significant results in bold.

## DISCUSSION

This study explored the impact of early-life EBV serology on oral HPV infection outcomes during the first 3 years of life. As expected, most children experienced a decline in maternal EBV antibodies over time. By the age of 3, the seroprevalence of EBV among children was approximately 17.2%. A significant protective factor against early-life EBV seropositivity was the father’s education level. However, no clear associations were found between EBV serology and oral HPV infections in children.

Transplacental transfer of maternal IgG antibodies from the mothers to their offspring during pregnancy protects the infants from infections until they mature their immune system (44). After birth, the transfer of maternal antibodies continues through breast milk and the IgA antibodies in it (44). Both HPV and EBV have been detected in breast milk; however, the clinical relevance of these findings remains poorly understood, and this transmission route is not typically considered essential (45, 46). Maternal antibodies typically decay within 6 to 12 months (44), with previous studies indicating that EBV-specific maternal antibodies generally decline by 8 months (47, 48). In our study, 91.4% of the children experienced EBV antibody decay, with significant correlations observed between the children’s EBV antibody levels at each time point and their mother’s corresponding baseline antibody levels, lasting up to the 6-month visit. Among the 220 children who remained antibody-negative after this decline, the average time to vanishing was 11.5 months, slightly exceeding previous estimates. Those who seroconverted subsequently experienced vanishing of maternal antibodies in 10.3 months (the mean of all 11.3 months).

The seroprevalence of EBV in children varies greatly, from 20 to 80%, depending on the age, geographic location, as well as the socioeconomic status (2). Higher seroprevalences are typically in the developing countries and in areas where EBV-associated cancers are endemic, such as Sub-Saharan Africa and Southeast Asia (49, 50). In developed countries, EBV seroconversion typically occurs in two peaks: the first between ages 2 and 4 and the second during adolescence up to age 20 (49, 51). Seroprevalence estimates for healthy 3-year-old children in the UK, Iran, and China are around 60% (52–55). A prior Finnish study reported EBV seroprevalence of 13.3% in children under 2 years and 46.3% in children aged 2 to 10 years (56). In our study, EBV seropositivity at 36 months was 17.2%, aligning with previous Finnish findings (56). These results together suggest that primary EBV infections are relatively uncommon in early childhood in Finland. However, since some children stayed seropositive throughout follow-up, their primary infection likely occurred close to the time of maternal antibody waning, indicating that early seroconversions can occur, potentially within the 1st year of life. Early childhood infections under the age of 1 are most likely acquired through close contact with family members, such as parents or older siblings. Since EBV can reactivate, a latently infected parent may serve as a source of transmission, and viral shedding among healthy carriers of EBV has been documented (57). In Finland, children typically enter daycare after 9 months of age due to parental leave policies, and some remain at home until age 3 through a home care allowance. This extended home care may reduce early exposure to infections like EBV. However, we lack data on viral shedding in family members and the exact timing of daycare entry, limiting our ability to assess infection sources. Notably, Fig. 1 shows an increase in EBV-IgG antibodies around 12 months, possibly reflecting increased exposure following daycare entry. It is interesting to note that the seroprevalences in highly endemic areas for EBV-related diseases are typically much higher than in this Finnish study, indicating that children in the highly endemic areas are exposed to the virus earlier in life (50).

Risk factors for early EBV infection include sharing a bedroom, parental education, caregiver smoking, breastfeeding, and siblings count (52, 58–61). Our study did not find a significant association between EBV seropositivity at ages 2 to 3 and passive smoking, family size, breastfeeding for at least 6 months, or maternal education. However, paternal education emerged as a significant protective factor. Even minimal education beyond basic education reduced risk (OR range of 0.09 to 0.16), with an academic degree offering the highest protection (OR 0.06). This aligns with earlier findings that higher household education level seems to be a protective factor for EBV seropositivity among children (59, 61), with an increased protective effect if both parents were educated (52). Yet, our results are in line with higher paternal education correlating with lower EBV seropositivity among children, though we did not observe the same effect for maternal education. The reason for this finding could be due to a possible link between higher education and a higher standard of living, and apparently, the biggest factor being particularly the level of paternal education. However, it is important to note that the number of fathers was relatively low, *n* = 89, and therefore it is inappropriate to draw strong conclusions.

We hypothesized that EBV antibody levels in early childhood could influence oral HPV infection outcomes, increasing the risk of persistence. However, no clear associations were found. Only high EBV EA-D IgG antibody levels at the 1-month visit correlated with oral HPV incidence and clearance, but this association did not persist at later time points. EA-D is an antigen of the lytic cycle and therefore a marker of recent or reactivated EBV infection (6, 62), and high titers of this antigen are often linked with EBV-associated diseases (63). However, elevated EA-D titers did not influence the HPV persistence infections in our study, and thus, our hypothesis was not supported. It is noteworthy that the number of significant associations (*n* = 3; 8%) is close to the expected false-positive rate under a 5% significance threshold, suggesting that some findings may be due to chance and should be interpreted with caution.

The strength of our study is the relatively long 36-month follow-up of 283 children from birth, with up to six visits to investigate EBV serology and oral HPV status during the first 3 years of life. To our knowledge, this is the first long-term study exploring this relationship. However, limitations include potential reporting bias from questionnaires, missing oral/oropharyngeal EBV DNA status, and the present EBV data based solely on serology and, furthermore, some missing blood samples.

### Conclusions

Most children experienced the vanishing of the maternal EBV antibodies. EBV seropositivity at ages 2 and 3 matched earlier Finnish estimates. Paternal education was a significant protective factor against early EBV seropositivity. No strong correlations were found between early-life EBV antibody levels and the longitudinal oral HPV infection outcomes. Long-term investigation of both EBV serology and local EBV DNA is needed to clarify their relationship with HPV infections. While early EBV infections and elevated antibody levels may contribute to EBV-related malignancies, their role in oral HPV infections remains unclear. Continued monitoring into early adulthood is essential to uncover potential long-term impacts.

## Data Availability

The data generated in this study are part of an ongoing longitudinal project and cannot yet be publicly released due to study governance and participant confidentiality considerations. Access to de-identified data may be granted upon reasonable request to the study principal investigator (K.L.), subject to data use agreements and institutional approval.
